# Severe mortality impact of the 1957 influenza pandemic in Chile

**DOI:** 10.1111/irv.12439

**Published:** 2017-03-31

**Authors:** Gerardo Chowell, Lone Simonsen, Rodrigo Fuentes, Jose Flores, Mark A. Miller, Cécile Viboud

**Affiliations:** ^1^ Georgia State UniversityAtlantaGeorgiaUSA; ^2^ Fogarty International CenterNational Institutes of HealthBethesdaMDUSA; ^3^ George Washington UniversityWashington DCUSA; ^4^ University of CopenhagenCopenhagenDenmark; ^5^ Ministerio de SaludSantiagoChile; ^6^ The University of South DakotaVermillionSDUSA; ^7^ Universidad de ChileSantiagoChile

**Keywords:** 1957 influenza pandemic, baseline mortality rates, Chile, excess mortality rates, latitude, reproduction number, transmissibility

## Abstract

**Introduction:**

Epidemiological studies of the 1957 influenza pandemic are scarce, particularly from lower‐income settings.

**Methods:**

We analyzed the spatial–temporal mortality patterns of the 1957 influenza pandemic in Chile, including detailed age‐specific mortality data from a large city, and investigated risk factors for severe mortality impact across regions.

**Results:**

Chile exhibited two waves of excess mortality in winter 1957 and 1959 with a cumulative excess mortality rate of 12 per 10 000, and a ~10‐fold mortality difference across provinces. High excess mortality rates were associated with high baseline mortality (*R* ^2^=41.8%; *P*=.02), but not with latitude (*P*>.7). Excess mortality rates increased sharply with age. Transmissibility declined from *R*=1.4‐2.1 to *R*=1.2‐1.4 between the two pandemic waves.

**Conclusions:**

The estimated A/H2N2 mortality burden in Chile is the highest on record for this pandemic—about three to five times as severe as that experienced in wealthier nations. The global impact of this pandemic may be substantially underestimated from previous studies based on high‐income countries.

## 1 Introduction

Prediction of the mortality impact of future pandemics is essential to ongoing pandemic preparedness efforts, but is hampered by a limited number of historical events available for study.^1^ In particular, few quantitative studies have documented the transmissibility and impact of the 1957 A/H2N2 influenza pandemic, and detailed mortality datasets are limited to economically developed nations of the Northern Hemisphere.^2^, ^3^, ^4^ Detailed age‐specific estimates are available for the United States and Canada only.^2^, ^5^, ^6^ Recently, we conducted a multinational mortality study using annualized data, which placed the burden of the 1957‐59 pandemic at 4.0 excess respiratory deaths per 10 000 population globally,^7^ a moderate estimate relative to the devastating 1918 pandemic,^8^ but up to 10 times greater than the 2009 pandemic.^9^ We noted, however, a 70‐fold difference in mortality estimates between countries during the 1957‐59 pandemic period, with Chile faring the worst.^7^ Importantly, development indicators only explained a fraction (35%‐77%) of the observed variation in country‐level pandemic burden estimates.^7^

Here, we follow up on our global analysis to explore pandemic patterns in Chile in more details using refined time series mortality data and models.

The first local outbreaks of pandemic influenza A/H2N2 virus activity were reported in continental China during February‐March 1957, and the emerging virus quickly reached Hong Kong and other parts of Asia in a matter of weeks.^10^ The pandemic reached Europe in June 1957 and South American countries of the Pacific coast, New Zealand, and South Africa in July of that year.^10^, ^11^ In the United States, sporadic outbreaks were reported during June‐August 1957, but widespread transmission was not observed until September as schools reopened. In this study, we collected and analyzed archival epidemiological data from Chile, a low‐income country at the time that was hit particularly hard.^7^ We characterize geographic variation in influenza‐related mortality patterns across Chilean provinces in the first 3 years of pandemic virus circulation and investigate age mortality patterns and transmissibility estimates.

## 2 Material and Methods

### 2.1 Epidemiological data sources

#### 2.1.1 National‐ and Province‐level Vital statistics, 1953‐1959

Chile had a population size of 6.9M in 1957 and was an emerging economy with excellent vital statistics records, a life expectancy at birth of just under 60 years, and a high infant mortality rate of 110 deaths per 1000 live births.^12^

We obtained monthly national all‐cause and respiratory mortality statistics for Chile from 1953 to 1959 from the Statistical Yearbooks of Chile. The choice of these study years allowed for estimation of a pre‐pandemic mortality baseline, and covers two waves of pandemic influenza A/H2N2 activity in 1957‐59.

To further explore geographic variation in pandemic‐related mortality in Chile, we also compiled monthly all‐cause mortality statistics from 1953 to 1959 for the 25 administrative provinces^12^ (Fig S1 in Appendix). Of note, because Chile has undergone changes in administrative divisions over the years, the geographic areas referred to in our study only approximately translate to contemporary administrative divisions in this country.

We also obtained province‐level population size estimates and infant mortality rates in 1956.^12^ We compiled latitude coordinates for province‐specific population centers to explore pandemic timing and impact along Chile's latitudinal gradient (17°S to 56°S^13^).

#### 2.1.2 Individual death certificates from the city of Concepcion, 1953‐1959

Given the lack of age detail in the official province‐level vital statistics, separate data sources were used to study the age patterns of pandemic mortality. The City of Concepcion, located in the southern central part of Chile, is the third largest city in this country and has preserved unique records of individual death certificates.^13^ We manually retrieved from the city's civil registry all 15 736 individual death certificates for the period of January 1953‐December 1959. For each record, we tabulated the age, cause, and date of death. We then created weekly all‐cause and respiratory mortality time series stratified into six age groups (<5 years, 5‐14 years, 15‐24 years, 25‐49 years, 50‐64 years, and >=65 years). We used a broad definition for respiratory deaths which included deaths from influenza, pneumonia, bronchopneumonia, or bronchitis as underlying causes. Less than 1% of records lacked age or cause of death information.

We obtained age‐specific population data for Concepcion from the Instituto Nacional de Estadísticas for year 1955 (total population of 170 457).

#### 2.1.3 Weekly mortality counts, Santiago and Concepcion, 1956‐1957

In order to characterize the reproduction number of the pandemic, we also obtained weekly all‐cause and respiratory mortality counts for 1956‐57 for the capital city, Santiago, from an official report.^14^, ^15^ Based on daily individual death certificates collected for Conception, we compiled weekly mortality time series for this city as well.

#### 2.1.4 Review of local newspapers

To support the identification of pandemic periods in mortality data, we gathered anecdotal information on the temporal course and severity of the pandemic by reviewing the most prominent daily newspapers published in Santiago (*El Mercurio,* Central Chile), Antofagasta (*El Mercurio de Antofagasta,* Northern Chile), and Concepcion (*El Sur, Southern Chile*) during 1957‐59. We identified 11 issues of *El Mercurio*, 25 issues of *El Mercurio de Antofagasta*, and 17 issues of *El Sur* that contained reports relating to the pandemic.

### 2.2 Statistical analysis

#### 2.2.1 Excess mortality estimates

To quantify the mortality burden associated with the influenza pandemic, we estimated the cumulative number of deaths occurring in excess of a model baseline during the period 1957‐59. Because mortality fluctuates seasonally throughout the year during this time period in Chile, we fit cyclical regression models with temporal trends and harmonic terms^16^, ^17^, ^18^ to monthly mortality in the pre‐pandemic period 1953‐56 to estimate baseline mortality. Pandemic periods were defined separately for each study population as the months when all‐cause or respiratory mortality exceeded the upper 95% confidence limit of the baseline. The absolute mortality burden of the pandemic was estimated as the sum of excess deaths during each pandemic period of 1957‐1959.

We also calculated the relative mortality burden of the pandemic, defined as the ratio of excess mortality during the pandemic periods over the expected baseline mortality for these periods. This relative approach facilitates comparison between countries, regions, and age groups with different background mortality risks.^2^, ^17^ Further, we performed sensitivity analyses to check the robustness of our excess mortality estimates to the choice of the mortality baseline and epidemic periods (Supplement).

#### 2.2.2 Geographic patterns

We analyzed geographic variation in timing and severity of the pandemic across the 25 provinces of Chile (Fig S1 in Appendix). For each geographic area, pandemic peak timing was defined as the month with maximal all‐cause mortality elevation in each winter during 1957‐1959. Severity was defined as absolute and relative rates of excess mortality for each pandemic wave during 1957‐1959, and cumulatively. We used univariate analyses and multivariate stepwise regression models to explore the association between province‐level estimates of peak timing and severity, and covariates such as latitude, population size, baseline and infant mortality rates.

#### 2.2.3 Estimation of transmission characteristics (reproduction number)

The reproduction number is an important parameter for pandemic preparedness as it quantifies the transmissibility potential of a pandemic virus and directly informs the strength of mitigation efforts required to interrupt transmission. Reproduction number estimates were derived from the weekly growth rate in respiratory deaths^19^, ^20^ in the two large cities included in our study, Santiago and Concepcion.

The pandemic growth rate was estimated by fitting an exponential function to the increase in weekly number of respiratory deaths at the start of each pandemic wave,^21^ assuming exponentially distributed latent and infectious periods or a fixed generation interval^19^ of three or four days.^22^, ^23^ We also tested the robustness of estimates to the choice of mortality outcomes, ranging from highly specific (excess respiratory deaths) to highly sensitive (all‐cause deaths).

## 3 Results

### 3.1 Newspaper information

The earliest reports of localized pandemic activity were on July 24, 1957, in Valparaiso, a large seaport north of Santiago, and influenza outbreaks were reported in Santiago 2 days later. The introduction of the pandemic virus was traced back to a US Navy vessel originating from an American port that had stopped in Valparaiso; an influenza‐like‐illness outbreak had been reported among the crew. Reports of a recrudescent influenza wave in September and October of 1959 were published in all three newspapers. In contrast, there was no mention of pandemic activity in the winter of 1958.

### 3.2 Geographic patterns, pandemic waves, and excess mortality

**Figure 1:**
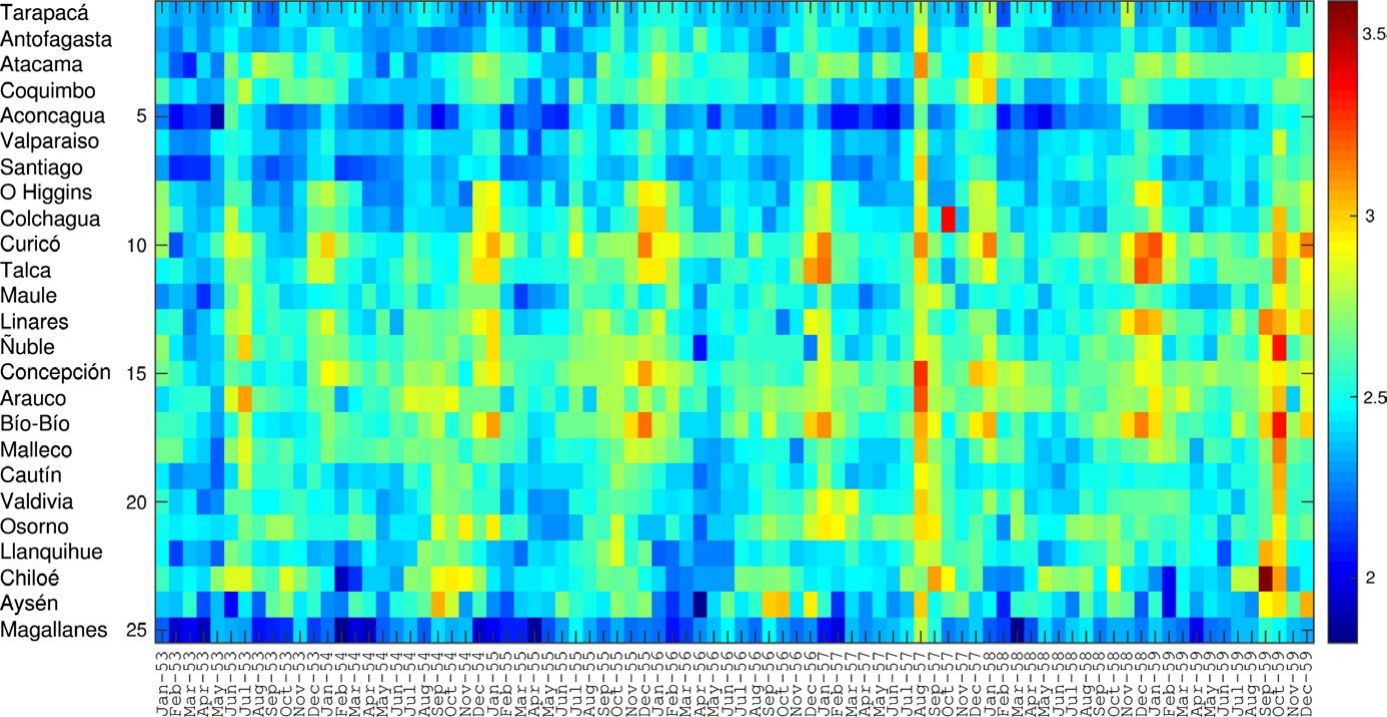
Temporal evolution of all‐cause mortality rates per 10 000 people during 1953‐1959 across 25 provinces of Chile, sorted in geographic order from north to south of Chile. For visualization purposes, the time series are log‐transformed

**Figure 2:**
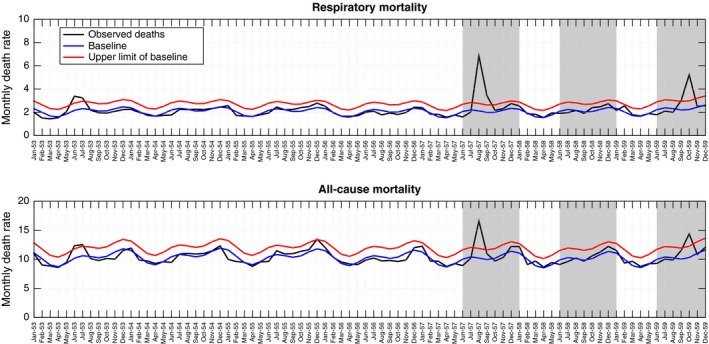
Monthly time series of all‐cause and respiratory mortality per 10 000 people in Chile, 1953‐1959 (black curve). Shaded areas highlight three winter periods (Jun‐Dec) during 1957‐1959. The Serfling seasonal regression model baseline (blue curve) and corresponding upper limit of the 95% confidence interval of the baseline (red curve) are also shown

Consistent with newspaper reports, mortality models indicate the occurrence of winter waves of pandemic‐related excess mortality in August‐October 1957 and September‐November 1959 in Chile (Figures 1, 2). Excess mortality estimates for the main pandemic wave in 1957 were 6.9 per 10 000 for all‐cause mortality and 6.7 per 10 000 for respiratory mortality. The second pandemic wave in 1959 was less severe with an estimated 5.1 and 3.2 excess deaths per 10 000 for all‐cause and respiratory diseases, respectively. No significant elevation in mortality was detected from national mortality time series during the winter of 1958. Across the entire pandemic period 1957‐1959, the cumulative mortality impact in Chile was 12.0 excess all‐cause deaths per 10 000, corresponding to ~8280 excess deaths or ~0.1% of the entire population.

Province‐level all‐cause mortality time series showed remarkable spatial synchronicity, with excess mortality occurring in August 1957 in 22 provinces, in September 1957 in 2 provinces, and lack of excess mortality in one province (Figure 1 and Appendix). Excess all‐cause mortality rates ranged from 0 to 23.4 per 10 000 across provinces for the main pandemic wave in 1957, with the provinces of Colchagua and Concepcion faring the worse (Table 1).

**Table 1:** Estimates of excess mortality rates attributable to pandemic influenza based on all‐cause mortality rates across 25 provinces of Chile. Excess mortality estimates for each wave were computed using a seasonal regression model applied to monthly mortality and presented as rates per 10 000 as described in the text. Provinces are sorted by latitude from north to south Chile

Provinces	Jul‐Dec 1957	Jun‐Dec 1958	Jun‐Dec 1959	Cumulative excess mortality rates, 1957‐1959
Tarapacá	4.2	4.9	6.3	15.4
Antofagasta	7.2	0.0	0.0	7.2
Atacama	8.1	0.0	0.0	8.1
Coquimbo	3.6	0.0	0.0	3.6
Aconcagua	7.3	3.0	0.0	10.3
Valparaíso	4.8	0.0	4.2	9
Santiago	8.4	1.8	2.6	12.8
O' Higgins	5.8	0.0	0.0	5.8
Colchagua	23.4	0.0	13.8	37.2
Curicó	7.6	0.0	0.0	7.6
Talca	5.7	4.5	5.6	15.8
Maule	4.0	0.0	4.5	8.5
Linares	0.0	4.2	5.1	9.3
Ñuble	3.6	0.0	10.6	14.2
Concepción	10.6	0.0	0.0	10.6
Arauco	7.4	0.0	0.0	7.4
Bío‐Bío	5.8	0.0	10.7	16.5
Malleco	6.6	0.0	6.1	12.7
Cautín	7.4	0.0	4.7	12.1
Valdivia	5.1	0.0	5.3	10.4
Osorno	4.6	0.0	0.0	4.6
Llanquihue	3.2	0.0	3.9	7.1
Chiloé	5.4	0.0	22.6	28
Aysén	5.2	0.0	7.0	12.2
Magallanes	5.4	0.0	0.0	5.4
National average	7.0	0.9	3.8	11.7

While no significant elevation in mortality rates was detected from national time series data in winter 1958, province‐level data revealed a minor pandemic wave in five Chilean provinces located in the north (n=3) and central regions (n=2) (Table 1). A recrudescent pandemic wave in winter of 1959 generated excess mortality rates in 15 provinces, with rates ranging from 0 to 22.6 excess deaths per 10 000 population. In nine provinces, the brunt of pandemic mortality impact was delayed until the winter of 1959. In particular, in Chiloé Island, the winter 1959 wave accounted for 80% of excess deaths attributed to the entire 1957‐1959 pandemic period (Table 1). There was no association between excess mortality rates in successive pandemic waves, so that the severity of the second wave was not predictive of the severity of the first wave (ρ=0.22; *P*>.3).

Cumulative all‐cause excess death rates for the 1957‐1959 pandemic period displayed a ~10‐fold difference across provinces, from 3.6 per 10 000 in Coquimbo to 37.2 per 10 000 in Colchagua (Table 1, Appendix). Stepwise multivariate regression identified baseline all‐cause mortality rates as the sole predictor of cumulative excess death rates (*R* 2=42%; *P*=.02). Absolute and relative cumulative mortality burden did not vary systematically with latitude (*P*>.7) or infant mortality rates (*P*>.6). Sensitivity analyses indicated that the ranking and range of province‐level excess mortality estimates were robust to more sensitive definitions of the epidemic periods and more conservative definitions of the baseline (correlation between methods in estimates of the 1957 and 1959 pandemic waves >0.92, *P*<.1, Table S1 and Fig S5).

### 3.3 Pandemic age mortality patterns (Concepcion)

**Figure 3:**
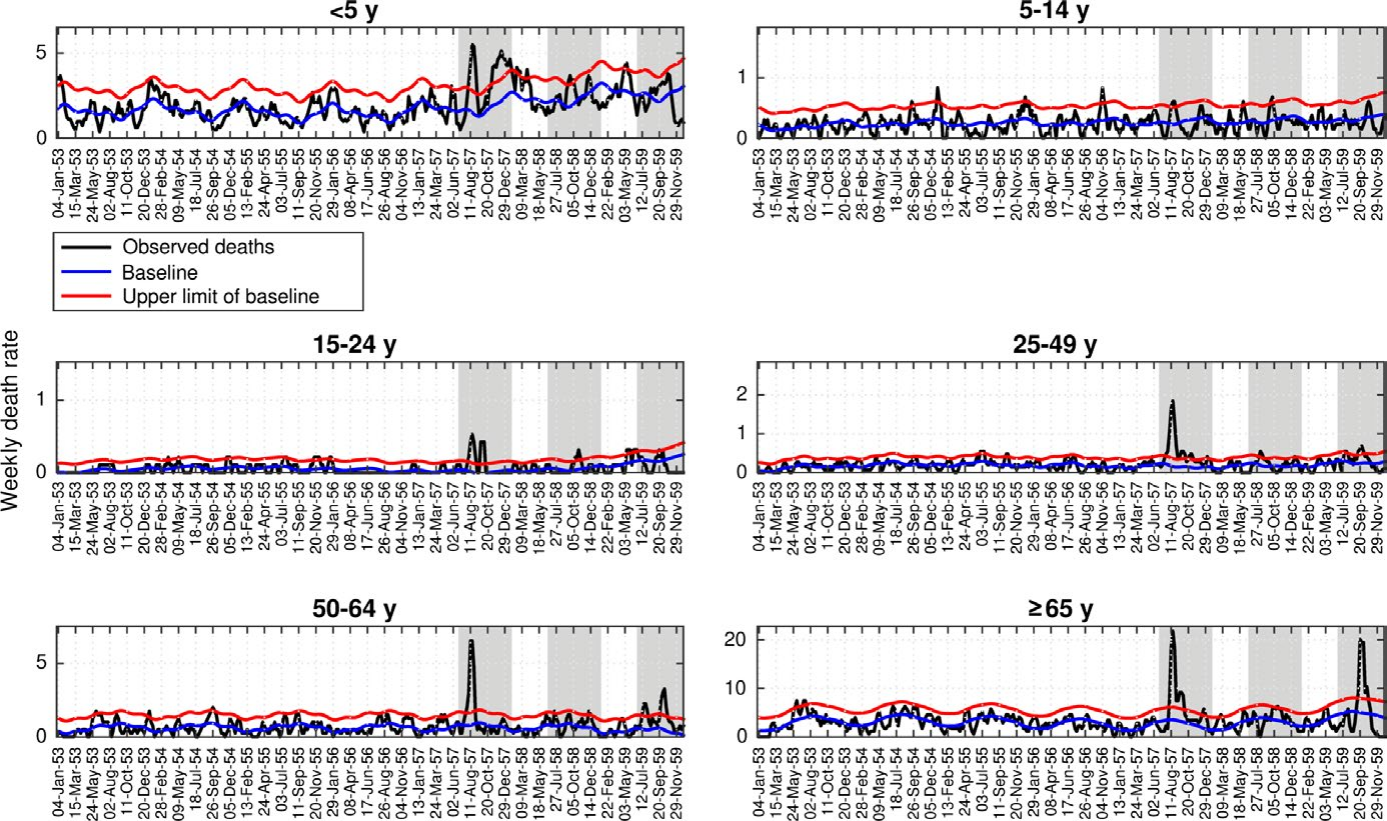
Age‐stratified weekly respiratory mortality rates per 10 000 in the city of Concepcion, Chile, 1953‐1959 (black curve). Shaded areas highlight three winter periods (Jun‐Dec) during 1957‐1959. The Serfling seasonal regression model baseline (blue curve) and corresponding upper limit of the 95% confidence interval of the baseline (red curve) are also shown. Excess deaths are above the upper limit of the baseline mortality curve calibrated using mortality levels prior to the 1957 influenza pandemic

Age‐specific analyses were limited to Concepcion due to data availability. Based on weekly mortality time series in Concepcion, the brunt of pandemic mortality occurred in July‐December 1957, while relatively minor mortality elevations were observed in winter 1958 and 1959 (Figure 3). All‐age excess respiratory death rates were estimated at 15.3, 1.5, and 4.3 per 10 000 for these three putative pandemic periods, for a total pandemic burden of 21.2 per 100 000. The all‐cause excess mortality estimate was similar (22.2 per 10 000 for the total pandemic period), indicating that Concepcion ranked relatively high among provinces with respect to pandemic severity.

Age‐specific excess mortality rates displayed a J‐shape in all three pandemic periods (Table 2). School‐age children of 5‐14 year old (yo) experienced the lowest cumulative excess death rates (1.8 excess respiratory deaths per 10 000), while seniors over 65 yo had the highest rates (166.3 per 10 000, or nearly 2% of all seniors dying). Age patterns were similar in all‐cause excess mortality rates (Table 2).

**Table 2:** Estimates of pandemic excess mortality rates, by pandemic wave, age groups, and cause of death, Concepcion, Chile, 1957‐59. Excess mortality estimates were based on a seasonal regression model applied to weekly respiratory and all‐cause mortality and presented as rates per 10 000 population

Pandemic period	<5 y	5‐14 y	15‐24 y	25‐49 y	50‐64 y	>=65 y	All ages
Respiratory excess death rates per 10 000							
Jul 1957‐Dec 1957	46.32	1.57	2.75	8.93	20.03	99.24	15.34
Jul 1958‐Dec 1958	7.14	0.23	0.19	0.16	2.30	6.40	1.51
Jul 1959‐Dec 1959	6.51	0.00	0.42	1.84	10.52	60.68	4.33
Total pandemic period	59.97	1.80	3.36	10.93	32.85	166.32	21.18
All‐cause excess death rates per 10 000							
Jul 1957‐Dec 1957	63.72	2.02	9.20	5.89	21.77	151.40	18.18
Jul 1958‐Dec 1958	7.25	1.03	0.55	0.17	1.12	25.15	0.91
Jul 1959‐Dec 1959	6.32	0.44	0.02	6.27	2.82	36.10	3.08
Total pandemic period	77.29	3.49	9.77	12.33	25.71	212.65	22.17

Mortality elevation relative to baseline (relative risk of death) varied from 5.7% to 113.4% during the pandemic periods based on all‐cause or respiratory mortality. Although seniors experienced the highest absolute excess death rates (Table 2), the relative risk of death was highest among young adults aged 15‐24 during the 1957 and 1958 pandemic waves. There was a shift toward older ages in the third pandemic wave in 1959, with 50‐ to 64‐year‐olds experiencing the highest relative burden.

### 3.4 Transmissibility estimates

**Figure 4:**
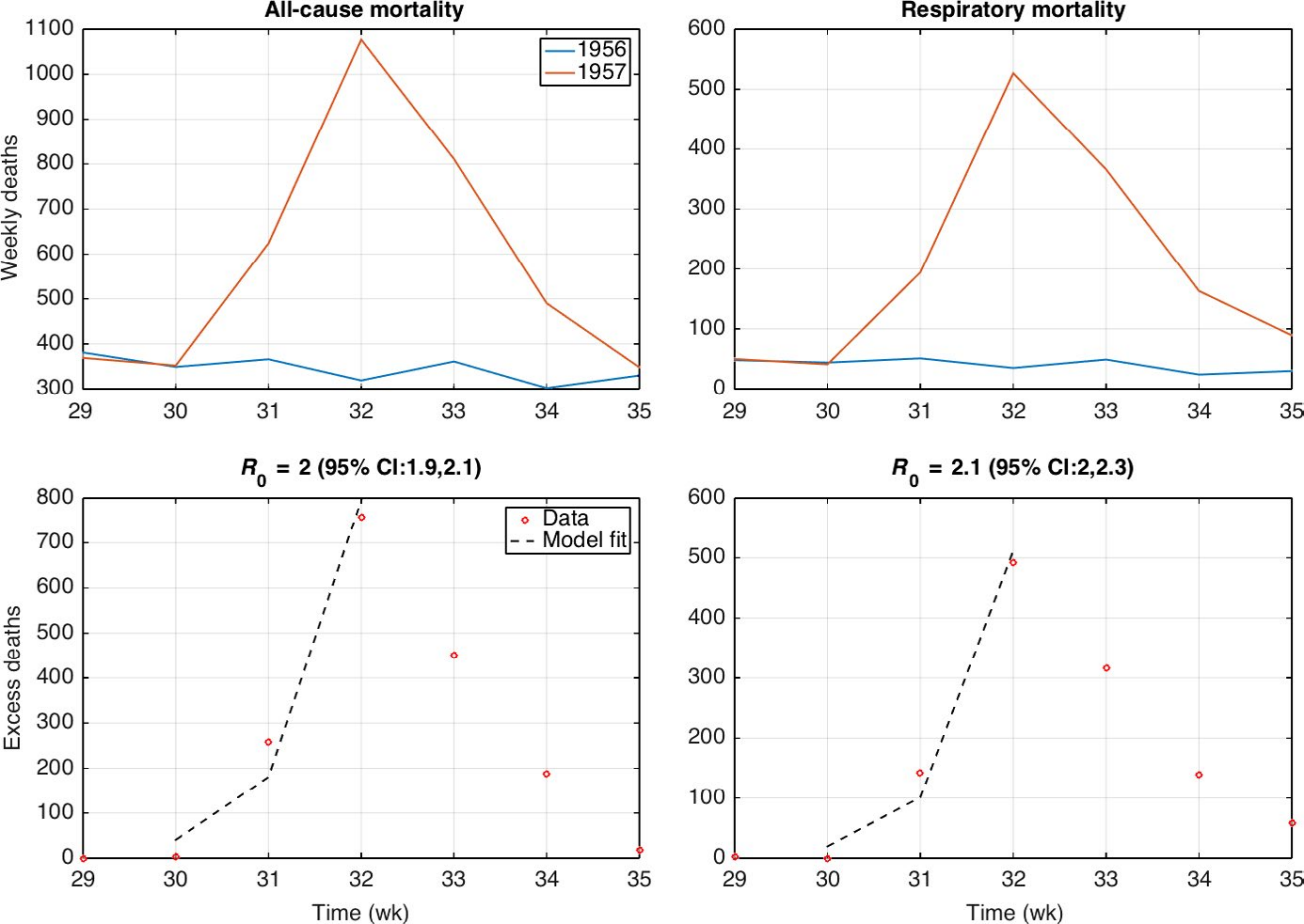
Weekly all‐age all‐cause and respiratory death counts in Santiago during weeks 29‐35 in 1957 and corresponding mortality baseline in 1956 during the same period (top). The basic reproduction number was estimated from the exponential growth fit to the initial phase of the excess mortality curve, which was computed by subtracting the 1956 baseline mortality curve to the 1957 pandemic period (bottom)

**Figure 5:**
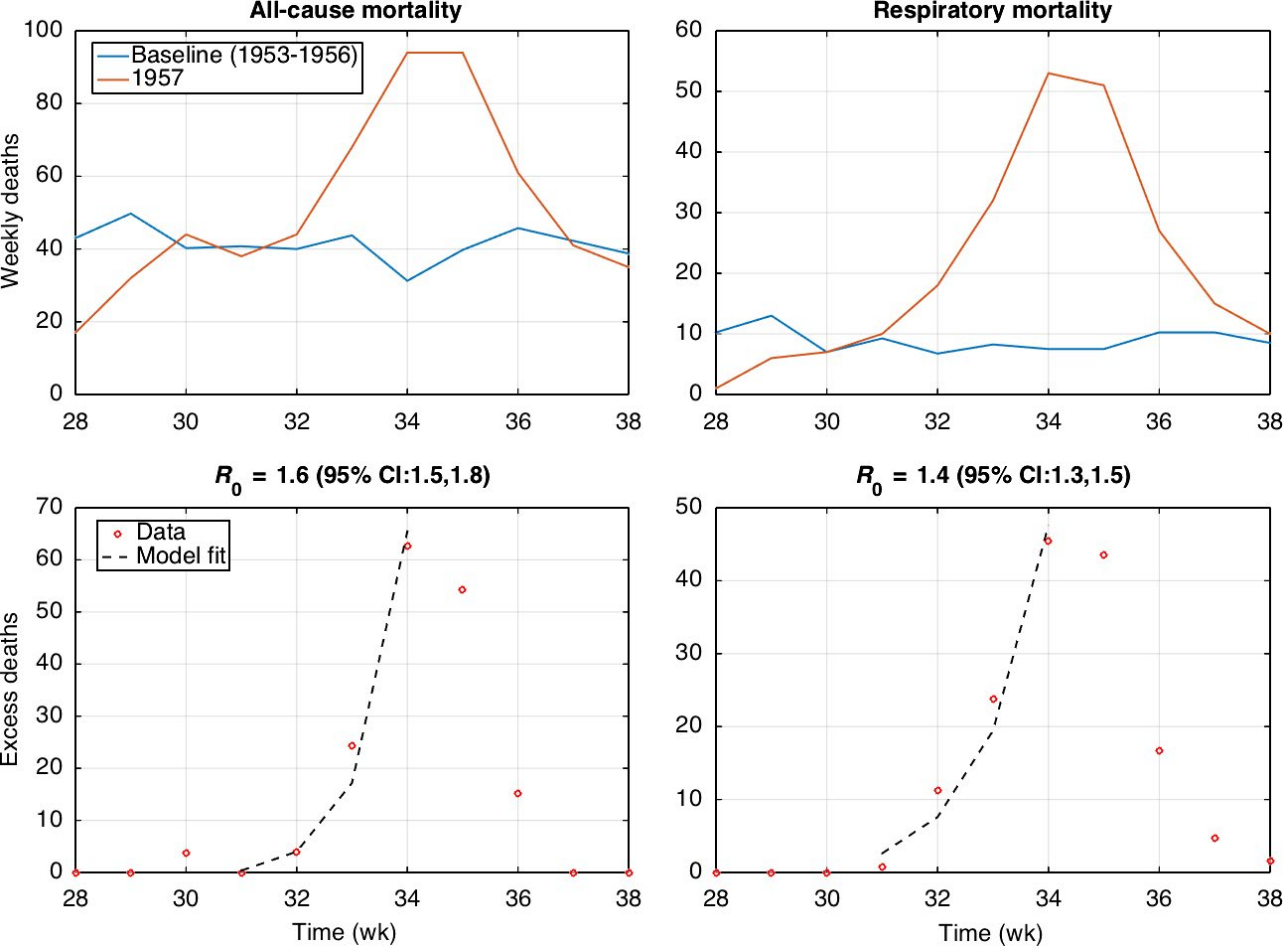
Weekly all‐cause and respiratory death counts in Concepcion during weeks 28‐38 in 1957 and the corresponding mortality baseline based on data from 1953 to 1957 during the same period (top). The basic reproduction number was estimated from the exponential growth fit to the initial phase of the excess mortality curve, which was computed by subtracting the baseline mortality curve to the 1957 pandemic period (bottom)

Reproduction number estimates ranged from 1.7 to 2.0 for the 1957 pandemic wave in Santiago, assuming a three‐day serial interval (Table 3; see Figures 4, 5 for the fit of the estimation procedure). Estimates were somewhat lower for the city of Concepcion, ranging from 1.4 to 1.7 (Table 3). Estimates were robust to use of different death outcomes, such as all‐cause mortality or excess mortality. The reproduction number for the 1959 recrudescent wave at 1.2‐1.3 in Concepcion was lower than for the first pandemic wave in 1957 (Table 3).

**Table 3:** Mean estimates and corresponding 95% confidence intervals for the transmissibility of the main wave of 1957 in Santiago, Chile, Mexico, assuming a serial interval of 3 or 4 d that is either exponentially distributed or fixed (delta distribution)

	3‐d serial interval		4‐d serial interval	
	Exp dist.	Delta dist.	Exp. Dist.	Delta dist.
Santiago, Chile				
Respiratory deaths	1.8 (1.7, 1.9)	2.0 (1.9, 2.1)	2.1 (2.0, 2.3)	2.5 (2.3, 2.8)
All cause	1.7 (1.7, 1.8)	1.9 (1.8, 2.0)	2.0 (1.9, 2.1)	2.3 (2.2, 2.5)
Concepcion, Chile				
1957 pandemic wave				
Respiratory deaths	1.4 (1.3, 1.5)	1.4 (1.3, 1.6)	1.6 (1.4, 1.7)	1.6 (1.4, 1.9)
All cause	1.6 (1.5, 1.8)	1.7 (1.5, 2.0)	1.9 (1.6, 2.1)	2.1 (1.8, 2.5)
1959 pandemic wave				
Respiratory deaths	1.2 (1.1, 1.3)	1.2 (1.1, 1.3)	1.3 (1.2, 1.4)	1.3 (1.2, 1.5)
All cause	1.2 (1.1, 1.3)	1.2 (1.1, 1.3)	1.3 (1.2, 1.3)	1.3 (1.2, 1.4)

## 4 Discussion

Analysis of extensive archival mortality statistics, death certificates, and contemporaneous newspaper reports reveals an unusually high excess mortality rate for the 1957‐59 influenza pandemic in Chile, estimated at 12 excess all‐cause deaths per 10 000, yielding the highest mortality estimates published for this pandemic. Two distinct pandemic waves occurred in winter 1957 and 1959, with highest pandemic‐related excess mortality rates occurring in the first wave in most provinces of Chile. In nine provinces however, the brunt of pandemic mortality impact was delayed to winter 1959. Most strikingly, pandemic‐related excess mortality rates for the period 1957‐1959 varied ~10‐fold across the 25 provinces of Chile; 42% of this variability was explained by differences in baseline mortality rates. Seniors experienced the highest pandemic excess death rates, with a shift in pandemic‐related mortality toward older ages in the later wave. Overall, the J‐shaped age patterns of mortality described here is in line with US and global studies of the 1957 pandemic.^2^, ^7^

We relied on excess mortality models applied to monthly all‐cause deaths to estimate pandemic burden at the province level, in line with prior studies.^24^, ^25^ Due to the severity of the 1957 pandemic in Chile, use of nonspecific mortality indicators such as monthly all‐cause mortality provides reliable estimates of influenza burden.^24^, ^26^ The validity of this approach is reinforced by the close agreement between all‐cause estimates and more specific estimates derived from respiratory deaths for national‐ or city‐level data, where both outcomes are available. Further, the near additivity of province‐specific mortality estimates (~8123 pandemic excess deaths), compared to the national estimates (~8280), supports the internal consistency of the excess mortality approach. Further, pandemic peak timing estimates derived from individual death certificates for Concepcion aligned with those derived from official all‐cause mortality statistics for the broader province, lending support to these summary statistics. We cannot rule out, however, that underreporting^8^ may have affected some of the province‐level differences evidenced here.

The onslaught of the pandemic A/H2N2 virus in Chile occurred during the coldest months of 1957, in line with pandemic patterns in other countries of the Southern Hemisphere, including New Zealand and South Africa, and ~5 months after the initial outbreak reports in China.^11^ This is in contrast to the 1918‐19 pandemic, where mortality was delayed until August 1919 in Chile, nearly 10 months after the lethal wave that characterized Northern temperate countries in September‐November 1918,^27^ and more than a year after the earliest available virologic evidence of influenza A/H1N1 virus circulation.^28^ Moreover, the timing of the main pandemic wave in winter 1957 was highly synchronous across Chilean provinces, in contrast to the lack of synchrony noted in the 1918‐20 pandemic in the same country.^13^ Increased domestic and international connectivity, and economic development, may have accelerated the spread of the 1957 A/H2N2 influenza pandemic in Chile, relative to the 1918 pandemic.

We found ~10‐fold differences in 1957‐59 pandemic excess death rates across provinces of Chile, which is commensurate with geographic variation reported for the 1918‐1920 influenza pandemic in the same country.^13^ To put these results in greater historical context, Chilean data indicate that the impact of the 1957‐59 pandemic was ~eightfold lower on average than that of the 1918‐20 pandemic (interquartile range 7.4‐13.2 vs 53.6‐97.2 deaths per 10 000 Wilcoxon test, *P*<.001),^13^ and 75‐fold higher than that of the 2009 pandemic.^9^ An association between baseline mortality and pandemic excess death rates was reported in 1918‐1920^13^ and 1957‐59 (this study), indicating that overall baseline death rates—which may be a proxy for degree of development—may account for ~40% of variability in pandemic severity at this scale. Underlying population risk factors and socioeconomic conditions^29^ were likely more important drivers of pandemic mortality rates than environmental factors. For instance, prior studies have suggested that indigenous groups could have been affected disproportionately from past influenza pandemics.^30^, ^31^, ^32^

The detailed mortality estimates provided here for Chile reinforce the findings from an earlier global study, pointing to a particularly high impact of the 1957‐59 pandemic in Chile.^7^ Other studies have estimated P&I excess mortality rates per 10 000 at 1.2‐1.8 in the United States for the first pandemic wave in 1957‐1958,^2^, ^3^, ^5^ 1.2 in Canada,^3^ and 1.1 in Germany,^33^ compared to 6.7 per 10 000 for Chile (Table 1). Reasons for the unusual severity of this pandemic in Chile remain unclear, and it is worth noting that Chile fared relatively well in contrast during the 1918 and 2009 pandemics.^9^, ^13^, ^29^

Analysis of death certificates from Concepcion allowed for the estimation of age‐specific burden. Excess mortality rates were lowest in school‐age children and increased monotonously with older ages, following a J‐shape reminiscent of US patterns.^2^, ^34^ This is indicative of lack of mortality protection among people older than 65 years, suggesting no prior exposure to antigenically related A/H2N2 viruses. In contrast, a Dutch study reported the presence of pre‐pandemic protective antibodies to A/H2 in seniors over ~70 years.^35^ The two studies are not necessarily inconsistent, as the size of the relevant age group was small in Concepcion and our study would have been underpowered to detect such an effect. Overall, it is generally accepted that senior sparing did not play a major role in the 1957 pandemic, in contrast to the 1918 and the 2009 pandemics in the United States^18^, ^26^ and Europe.^9^, ^36^, ^37^, ^38^

Based on weekly city‐level respiratory deaths, transmissibility of 1957 pandemic wave was significantly higher in Santiago (*R*~1.7‐2.5) than in Concepcion (*R* ~1.4‐1.6), perhaps due to higher population density in the capital city. However, transmissibility estimates based on all‐cause mortality overlapped. We also found a lower *R* estimate for the 1959 pandemic wave in Concepcion (*R* ~1.2‐1.3), as would be expected with buildup of population immunity. Prior studies have estimated the mean reproduction number of the 1957 influenza pandemic in the range 1.5‐1.8 in England and Wales,^39^, ^40^ which aligns well with our estimates. For comparison, the reproduction number for the 1918‐20 pandemic ranges from 1.5 to 5.4 for community‐based settings^41^, ^42^ and from 2.1 to 7.5 in confined settings.^41^ Thus, the atypical severity of the 1957 pandemic in Chile does not stem from unusually high transmissibility.

Our study has several limitations. First, while both respiratory and all‐cause monthly mortality data were available at the national level from Statistical Yearbooks, only all‐cause mortality data were available at the regional level. Second, regional data were not stratified by age or gender, preventing further analyses of demographic shifts across regions. Due to the lack of laboratory confirmation, our excess mortality approach would not have been unable to distinguish elevation in mortality rates associated with other causes and coinciding with the pandemic period. For instance, RSV circulation among infants during the pandemic period cannot be ruled out and could have inflated our pandemic mortality estimates in this age group; however, it is unlikely RSV had major impact on estimates for older age groups. Further, review of the gray literature points to influenza as a very plausible etiology for excess mortality occurring in winter 1957 and 1959 in Chile, and these findings are in line with recrudescent waves reported in all major influenza pandemics.^38^ A final caveat relates to our excess mortality calculation approach, which was relatively simple, due to the lack of contemporaneous virologic surveillance and relatively weak temporal resolution of the mortality data. However, methods for estimating excess mortality data are not free of limitations.^43^ For instance, the excess methodology employed in our study does not guarantee that excess mortality estimates are additive by age group.^44^ Sensitivity analyses, however, indicate that our estimates are robust to modeling assumptions (Supplement).

In conclusion, our historical‐epidemiological analysis reveals unusually high mortality during the 1957‐59 influenza pandemic in Chile, the southernmost country of Latin America, with the highest excess mortality estimate published for this pandemic. We found a ~10‐fold difference in pandemic excess mortality rates between Chilean provinces, with overall baseline mortality rates explaining a moderate amount of variation (40%). Excess mortality rates increased with age, indicating a lack of measurable mortality sparing among senior populations. Overall, our study reinforces the importance of geographic variation in pandemic mortality burden between and within countries, echoing recent work on past pandemics.^7^, ^9^, ^29^ Such variability needs to be recognized, especially in light of WHO's anticipated inclusion of a severity indicator as an element of future pandemic responses. We recommend that pandemic severity assessment should be based on multiple “sentinel” countries representing several world regions representing a variety of health and income conditions.

## Conflict of Interest

The authors declare no conflict of interest relevant to this study.
