# Supplementary material for: Severe mortality impact of the 1957 influenza pandemic in Chile
Source: Influenza Other Respir Viruses. 2017 Mar 31;11(3):230–9. doi: 10.1111/irv.12439 (PMC5410718; doi:10.1111/irv.12439)
Supplement: Supplementary file 1 [file IRV-11-230-s001.docx]

**Appendix**

**Figure S1.** Map of Chile with 25 contiguous administrative provinces in 1957. Chile covers a long and narrow strip between the Andes mountains to the east and the Pacific Ocean to the west, ranging between latitude 17° and 56°S and longitudes 66° and 76°W.

**
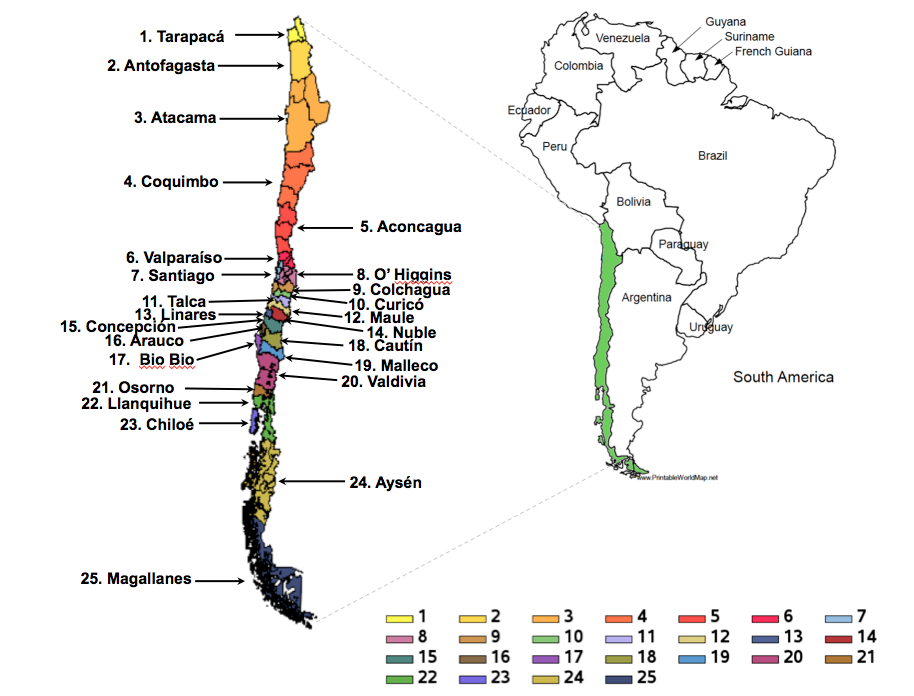
**

**Figure S2**. Monthly time series of all-cause mortality per 10,000 people in 25 Chilean provinces, 1953-1959 (black curve). The Serfling seasonal regression model baseline (blue curve) and corresponding upper limit of the 95% confidence interval of the baseline (red curve) are also shown.


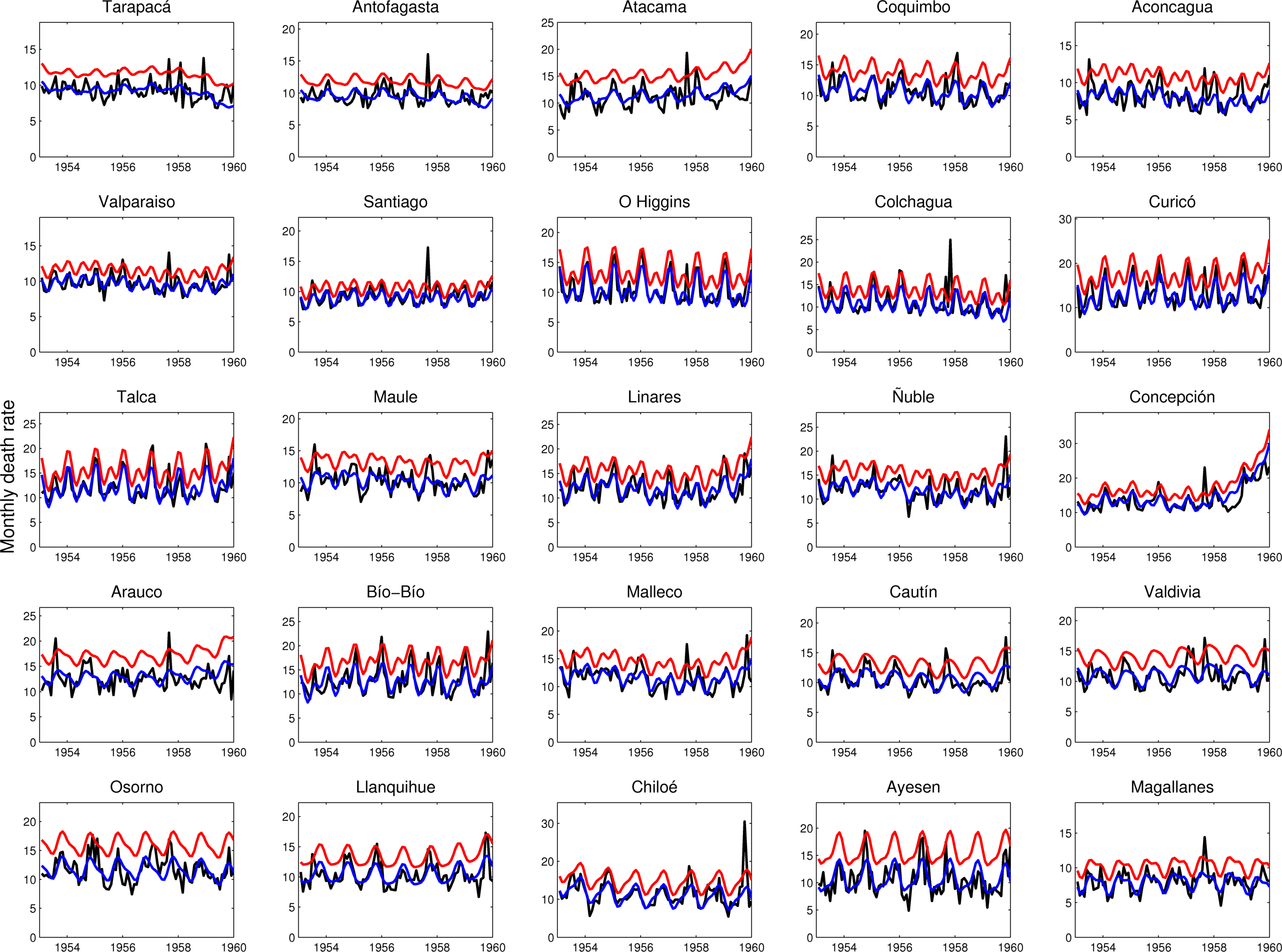


**Figure S3.** Monthly time series of all-cause mortality counts across 25 Chilean provinces in 1957. Red crosses indicate the peak mortality impact of the pandemic in August and September, 1957.


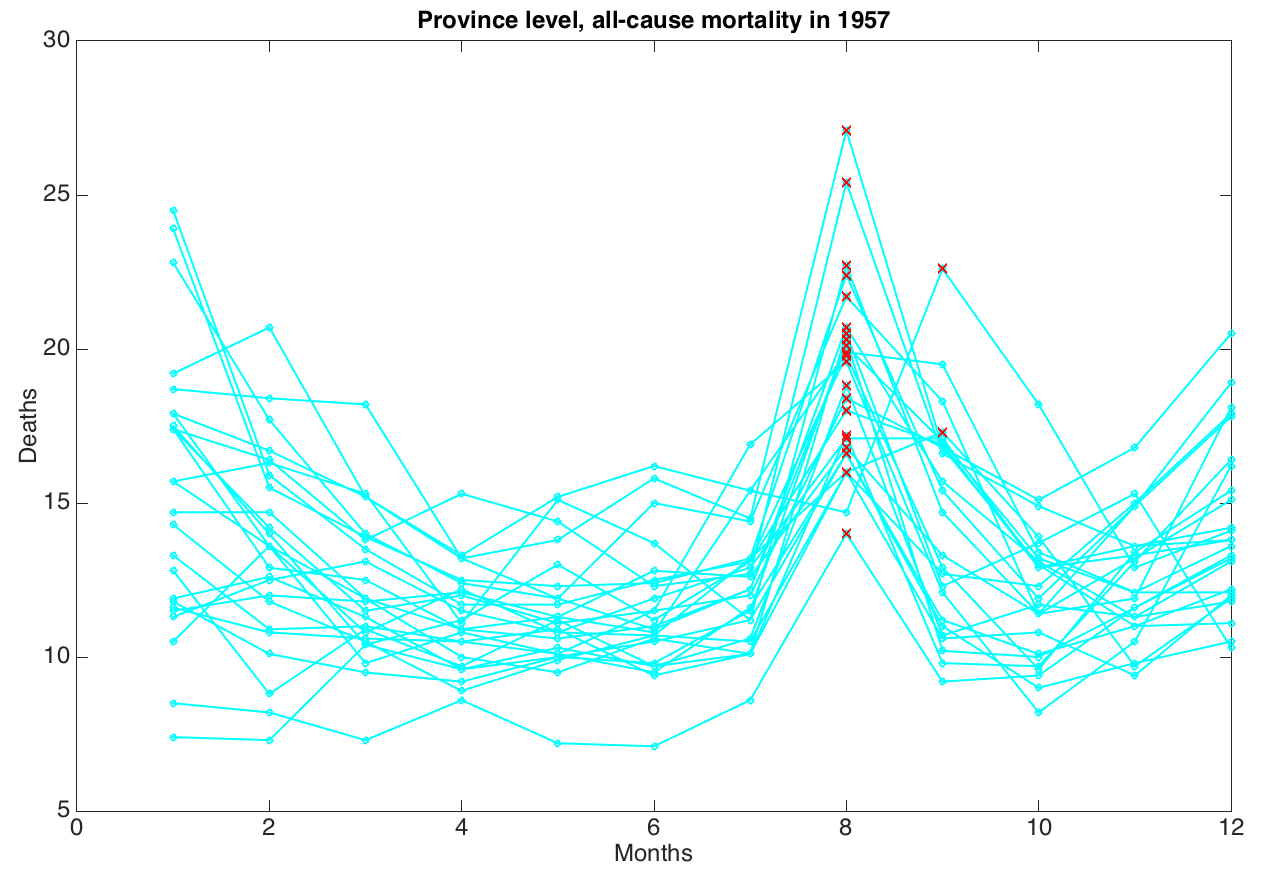


**Figure S4.** Excess mortality rates per 10,000 across 25 provinces of Chile according to pandemic periods (Jul-Dec 1957; Jun-Dec 1958; Jun-Dec 1959) in geographic order from north to south Chile. Excess deaths are above the upper limit of the baseline mortality curve calibrated using all-cause monthly mortality levels prior to the 1957 influenza pandemic.


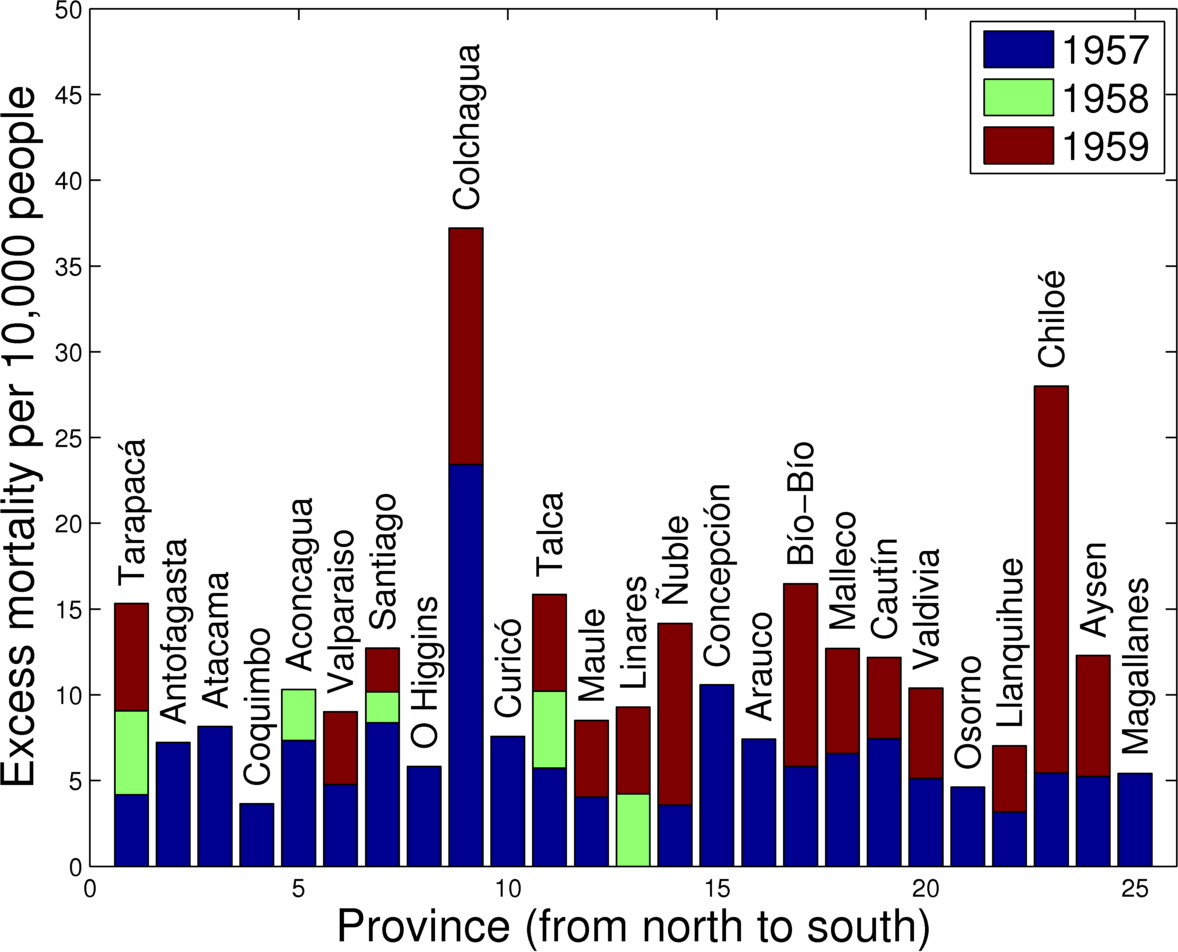


**Supplementary Figure 5**: Scatter plot of province-level pandemic burden estimates considering different excess mortality approaches. The approach taken in the main analysis (a) considers independent epidemic periods in each province, defined as months of 1957-59 when the observed province-specific all-cause mortality exceeds the upper 95% CI of a seasonal baseline model and excess mortality is defined as the observed minus baseline all-cause mortality rate (x-axis on all 3 panels). Two alternative approaches are explored, in which (blue dots, y-axis) excess is defined as the difference between observed and the upper 95% CI of the baseline or (red dots, y-axis), pandemic periods are fixed nationally (Aug-Oct 1957 and Sep-Nov 1959; no pandemic period in 1958). This plot indicates strong agreement between these 3 methods, lending support to the robustness of our burden estimates.

**Supplementary Table S1**: Sensitivity analyses regarding the estimation of all-cause excess mortality rates at the province level. The approach taken in the main analysis (a) considers independent epidemic periods in each province, defined as months of 1957-59 when the observed province-specific all-cause mortality exceeds the upper 95% CI of a seasonal baseline mode. In addition, excess mortality is defined as the observed minus baseline mortality rate. Two alternative approaches are explored, in which (b) excess is defined as the difference between observed and the upper 95% CI of the baseline or (c), pandemic periods are fixed nationally (Aug-Oct 1957 and Sep-Nov 1959). Note that by definition, no excess mortality is attributed to influenza in 1958 by method c). Further, method b) provides lower excess mortality estimates on average than the other two approaches, although the geographic patterns are similar. Suppl Fig 4 provides correlation plots, supporting high agreement between these approaches (Pearson rho > .90, P<0.05)).

| **Statistics across 25 provinces** | **1957** | | |  | **1958** | | |  | **1959** | | |
| --- | --- | --- | --- | --- | --- | --- | --- | --- | --- | --- | --- |
|  | **a) Main analysis** | **b) Excess is difference with upper 95% CI of baseline** | **c) Pandemic period is fixed at Aug-Oct 1957** |  | **a) Main analysis** | **b) Excess is difference with upper 95% CI of baseline** | **c) No pandemic period in 1958** |  | **a) Main analysis** | **b) Excess is difference with upper 95% CI of baseline** | **c) Pandemic periods is fixed at Sep-Nov 1959** |
| **Median excess mortality rate per 10,000** | 5.7 | 2.4 | 7.4 |  | 0 | 0 | 0 |  | 4.2 | 0.5 | 5.3 |
| **Range of excess mortality rates per 10,000** | [0 - 23.4] | [0 - 17.3] | [4.1 - 25.3] | | [0 - 4.9] | [0 - 2.4] | [0 - 0] |  | [0 - 22.6] | [0 - 13.7] | [0 - 22.6] |
